# Electrochemotherapy as an Alternative Treatment Option to Pelvic Exenteration for Recurrent Vulvar Cancer of the Perineum Region

**DOI:** 10.1177/15330338221116489

**Published:** 2022-07-27

**Authors:** Gregor Vivod, Nina Kovacevic, Maja Čemažar, Gregor Serša, Tanja Jesenko, Maša Bošnjak, Simona Kranjc Brezar, Sebastjan Merlo

**Affiliations:** 1 Department of Gynecological Oncology, 68196 Institute of Oncology Ljubljana, Ljubljana, Slovenia; 2 Medical Faculty Ljubljana, 37663 University of Ljubljana, Ljubljana, Slovenia; 3 Faculty of Health Care Angela Boškin, Jesenice, Slovenia; 4 Department of Experimental Oncology, 68196 Institute of Oncology Ljubljana, Ljubljana, Slovenia; 5 Faculty of Health Sciences, 68960 University of Primorska, Izola, Slovenia; 6 Faculty of Health Sciences, 37663 University of Ljubljana, Ljubljana, Slovenia; 7 Faculty of Pharmacy, 37663 University of Ljubljana, Ljubljana, Slovenia; 8 Medicical Faculty, 54765 University of Maribor, Maribor, Slovenia

**Keywords:** vulvar cancer, electrochemotherapy, pelvic exenteration, recurrent disease, surgery

## Abstract

**Objective**: Pelvic exenteration in women with recurrent vulvar carcinoma is associated with high morbidity and mortality and substantial treatment costs. Because pelvic exenteration severely affects the quality of life and can lead to significant complications, other treatment modalities, such as electrochemotherapy, have been proposed. The aim of this study was to evaluate the feasibility and suitability of electrochemotherapy in the treatment of recurrent vulvar cancer. We aimed to analyze the treatment options, treatment outcomes, and complications in patients with recurrent vulvar cancer of the perineum. **Methods:** A retrospective analysis of patients who had undergone pelvic exenteration for vulvar cancer at the Institute of Oncology Ljubljana over a 16-year period was performed. As an experimental, less mutilating treatment, electrochemotherapy was performed on one patient with recurrent vulvar cancer involving the perineum. Comparative data analysis was performed between the group with pelvic exenteration and the patient with electrochemotherapy, comparing hospital stay, disease recurrence after treatment, survival after treatment in months, and quality of life after treatment. **Results:** We observed recurrence of disease in 2 patients with initial FIGO stage IIIC disease 3 months and 32 months after pelvic exenteration, and they died of the disease 15 and 38 months after pelvic exenteration. Two patients with FIGO stage IB were alive at 74 and 88 months after pelvic exenteration. One patient with initial FIGO stage IIIC was alive 12 months after treatment with electrochemotherapy with no visible signs of disease progression in the vulvar region, and the lesions had a complete response. The patient treated with electrochemotherapy was hospitalized for 4 days compared with the patients with pelvic exenteration, in whom the average hospital stay was 19.75 (± 1.68) days. **Conclusion:** Our experience has shown that electrochemotherapy might be a less radical alternative to pelvic exenteration, especially for patients with initially higher FIGO stages.

## Introduction

Vulvar cancer is the fourth most common gynecologic cancer, with an incidence of 2.6 per 100 000 women per year.^1^ Historically, vulvar cancer has been and continues to be treated with radical vulvectomy and bilateral groin lymph node dissection or sentinel lymph node biopsy.^2^ Radiotherapy may be used as adjuvant therapy after initial surgery or as part of primary therapy for locally advanced diseases. According to guidelines, surveillance is recommended every 3 to 6 months for 2 years after initial treatment, then every 6 to 12 months for another 3 to 5 years, and then annually.^3^ Recurrent vulvar cancer occurs in an average of 24% of cases after primary treatment after surgery with or without radiation.^3^ Most recurrences occur locally near the surgical margins or in the contralateral lymph node groin region. The therapeutic modalities used depend on the localization, extent of recurrence, and prior radiotherapy or concomitant chemoradiotherapy.^4^

In oncologic terminology, pelvic exenteration refers to en bloc resection of the viscera and pelvic organs of the female reproductive tract. Pelvic exenteration is classified as anterior, posterior, and total. In anterior pelvic exenteration, the bladder is removed with or without urethra and formation of urinary diversio, ileal conduit, or continent urinary diversion with or without hysterectomy, with or without resection of vagina and perineum. Posterior pelvic exenteration involves the removal of the rectosigmoid colon and, in some patients, the anal canal with primary anastomosis or formation of an end colostomy with or without hysterectomy.^5^ Total pelvic exenteration is the removal of the internal reproductive tract, bladder, and rectosigmoid colon. Further subclassification is made regarding muscle levator ani as the point of resection. Supralevator pelvic exenteration is performed above the levator ani muscle (type 1), infralevator pelvic exenteration is performed to preserve or resect the levator ani muscle without vulvectomy (type 2) and pelvic exenteration with vulvectomy (type 3).^6,7^

Posterior pelvic exenteration with vulvectomy (type 3) is a generally accepted surgical indication for recurrent vulvar cancer previously treated with radio- or chemoradiotherapy.^7–9^ Pelvic exenteration has high mortality and morbidity rate. Due to improvements in preoperative planning, surgical techniques, and peri- and postoperative care, morbidity has decreased over the years, but surgical procedures still significantly affect patients' quality of life.^10–12^ Therefore, new therapeutic options are warranted.

Electrochemotherapy is a local ablative therapy in which the application of reversible electrical pulses to the tumor permeabilizes the cell membrane, allowing cytotoxic drugs to penetrate the cells.^13^ It is most commonly used to treat superficial tumors such as melanoma, sarcoma, squamous cell carcinoma, basal cell carcinoma, skin metastases from breast cancer, and others.^14,15^ It can also be used to treat deep-seated tumors such as primary hepatocellular carcinoma, unresectable colorectal liver metastases, or pancreatic cancer.^16–18^ It is conducted following standard operating procedures, and the method is now used in nearly 170 comprehensive cancer centers around the world.^15^

There are few papers describing the use of electrochemotherapy in the palliative treatment of gynecologic cancer, especially squamous cell carcinoma of the vulva.^19,20^ Safety and local efficacy following electrochemotherapy with bleomycin for locoregional cutaneous recurrences of vulvar carcinoma previously treated with chemotherapy, radiotherapy, and surgery or unsuitable for standard treatments have been demonstrated.^21–24^

Because pelvic exenteration severely compromises the quality of life and can lead to significant complications,^25^ other treatment modalities such as electrochemotherapy have been proposed.

To evaluate the feasibility and suitability of electrochemotherapy in the treatment of recurrent squamous cell vulvar cancer, we analyzed the treatment options, treatment outcomes, and complications in patients with recurrent squamous cell vulvar cancer of the perineum.

## Methods and Materials

### Study Design

A retrospective institutional-based analysis was performed for patients who underwent surgery for vulvar cancer from January 1, 2006 to May 31, 2021 (16-year interval) at the Institute of Oncology Ljubljana. The data collection and analysis were approved by the Medical Board of the Institute based on the positive opinion of Institutional Review Board and Ethical Committee (ERIDNPVO-0001/2020). Informed consent was waived due to the retrospective nature of the study based on approval. The patient data were obtained from the Institutional database Web Doctor 4.2.0 (Marand inženiring d.o.o., Ljubljana, Slovenia). The reporting of this study conforms to STROBE guidelines.^26^

### Patients and Data Collection

In the analysis of surgical procedures, all procedures except pelvic exenterations were excluded during the observed period in patients without the metastatic spread of the disease. Inpatient reports of 4 patients who had undergone pelvic exenteration were analyzed in terms of primary tumor characteristics, treatment history, details of the procedure, hospital stay, recurrence of disease after treatment, survival after treatment in months, and quality of life after treatment. One patient with recurrent vulvar carcinoma involving the perineum was referred to our institution in June 2021. After clinical evaluation and diagnostic imaging, the patient was presented to the Interinstitutional Tumor Board, which decided that electrochemotherapy was a safe and feasible treatment approach before possible posterior pelvic exenteration with abdomino-perineal excision for the purpose of neoadjuvant therapy. The tumor board consisted of medical oncologists, radiotherapists, and gynecologic oncologists. The electrochemotherapy protocol was approved by the Institutional Medical Board and the Slovenian National Ethics Committee (number 0120-262/2021/3). As an experimental, less mutilating treatment, electrochemotherapy was performed on a patient with recurrent vulvar cancer involving the perineum. Electrochemotherapy with bleomycin was performed according to the standard operating procedure.^15^ Briefly, the patient was under general anesthesia. Bleomycin was administered at a dose of 15 000 IU/m^2^ (Bleomicin Medac, Medac GmbH, Germany). Eight minutes after intravenous administration of the drug, electrical pulses were applied to the tumors in a way that all tumor nodules were covered including the safety margin of ∼1 cm. Hexagonal geometry needle electrodes were used and electrical pulses were generated by Cliniporator (Igea S.P.A., Italy). Altogether 10 applications of electric pulses were delivered.

After the procedure, comparative data analysis was performed between the pelvic exenteration group and the electrochemotherapy patient, comparing hospital stay, recurrence of disease after treatment, survival in months, and posttreatment quality of life.

Pelvic computed tomography (CT) and/or magnetic resonance imaging (MRI) and positron emission tomography-computed tomography (PET-CT) scans were performed preoperatively in all included patients to assess the extent of local metastatic disease and to rule out distant metastasis. All patients were previously informed of the purpose of the study, participated voluntarily, and provided signed, written informed consent. In addition, all patient details have been de-identified.

## Results

During the study period, 4 cases of pelvic exenteration for recurrence of vulvar cancer in the perineal region were identified in the database. Two patients underwent total pelvic exenteration and 2 underwent posterior pelvic exenteration. Patient and tumor characteristics are shown in Table 1, surgical characteristics in Table 2, and pain and quality of life in Table 3.

**Table 1.** Patients and Tumor Characteristics.

Patient	Age (year) at PE or ECT	FIGO vulvar cancer stage after primary treatment	Site of vulvar cancer recurrence	Histologic type	Previous irradiation	Type of treatment of vulvar cancer recurrence	Recurrence of disease after PE or ECT (months)	Survival after PE or ECT (months)
1	70	IIIC	Perineum, Introitus	SCC	Yes	TPE	3	Died (15)
2	51	IB	Perineum, Vagina	SCC	Yes	TPE	nr	Alive (74)
3	60	IIIC	Perineum	SCC	Yes	PPE	32	Died (38)
4	72	IB	Perineum	SCC	Yes	PPE	nr	Alive (88)
5	64	IIIC	Perineum	SCC	Yes	ECT	nr	Alive (12)

Abbreviations: FIGO, International Federation of Gynecology and Obstetrics; PE, pelvic exenteration; TPE, total pelvic exenteration; PPE, posterior pelvic exenteration; ECT, electrochemotherapy; nr, no recurrence; SCC, squamous cell carcinoma.

**Table 2.** Surgical Characteristics.

Patient	Type of treatment of vulvar cancer recurrence	Hospital stay (days)	Details of surgery	Plastic reconstruction	Surgical margins after PE or ECT	Revisional surgery
1	TPE	21	Removal of bladder with urethra, formation of urinary diversion (an ileal conduit), hysterectomy with resection of the vagina and perineum, Removal of rectosigmoid colon with formation of an end colostomy	VRAM	Free of malignancy	No
2	TPE	15	Removal of bladder with urethra, Formation of urinary diversion (an ileal conduit), Resection of the vagina and perineum (hysterectomy in the past), Removal of rectosigmoid colon with formation of an end colostomy	VRAM	Free of malignancy	No
3	PPE	21	Resection of distal urethra, perineum, Removal of rectosigmoid colon with formation of an end colostomy	Primary suturing	Free of malignancy	For rectovaginal fistula
4	PPE	22	Resection of the vagina and perineum (hysterectomy in the past), Removal of rectosigmoid colon with formation of an end colostomy	VRAM	Free of malignancy	No
5	ECT	4	ESOPE	–	Free of malignancy	No

Abbreviations: PE, pelvic exenteration; TPE, total pelvic exenteration; PPE, posterior pelvic exenteration; ECT, electrochemotherapy; VRAM, vertical rectus abdominis myocutaneous flap; ESOPE, European Standard Operating Procedures for Electrochemotherapy.

**Table 3.** Pain and Quality of Life.

Patient	Type of treatment of vulvar cancer recurrence	Pain described on the VAS scale 1 month after PE or ECT	Pain described on the VAS scale 6 months after PE or ECT	Pain described on the VAS scale 12 months after PE or ECT	Short-term complications after PE or ECT	Long-term complications after PE or ECT	Clavien-Dindo classification	CCI
1	TPE	3/10	3/10	5/10	Partial necrosis of VRAM	Pain, leakage of ileal conduit	III-b	48.5
2	TPE	3/10	2/10	0/10	Colostomy and ileal conduit handling problems	No long-term complications	I	8.7
3	PPE	2/10	0/10	0/10	Wound infection, Rectovaginal fistula	Leakage of colostomy	III-b	44.9
4	PPE	3/10	2/10	1/10	Necrosis of colostomy	No long-term complications	III-b	33.7
5	ECT	4/10	0/10	0/10	No short-term complications	No long-term complications	/	/

Abbreviations: PE, pelvic exenteration; TPE, total pelvic exenteration; PPE, posterior pelvic exenteration; ECT, electrochemotherapy; VAS, Visual Analogue Scale; VRAM, vertical rectus abdominis myocutaneous flap; CCI, comprehensive complication index.

All patients had squamous cell carcinomas and all were initially treated with surgery and radiotherapy. After primary treatment, 2 patients were classified as International Federation of Gynecology and Obstetrics (FIGO) vulvar carcinoma stage IIIC, and 2 patients were classified as stage IB. The mean age of patients with pelvic exenteration was 63.3 (± 3.76) years. In all patients, distant metastases were excluded and resections were radical. In all patients with posterior or total pelvic exenteration, a permanent proximal end colostomy was created. In 2 patients with total pelvic exenteration, an ileal conduit was created.

All patients with pelvic exenteration experienced short- or long-term complications, such as handling problems with colostomy and ileal conduit, pain, wound infections, rectovaginal fistulas, partial necrosis of the VRAM, and necrosis of the colostomy. One of the patients required reoperation because of a rectovaginal fistula. Quality of life was significantly impaired after surgery.

We observed recurrence of disease in 2 patients with initial FIGO stage IIIC disease 3 months and 32 months after pelvic exenteration, and they died of the disease 15 and 38 months after pelvic exenteration. Two patients with FIGO stage IB were alive at 74 and 88 months after pelvic exenteration.

**Figure 1.**
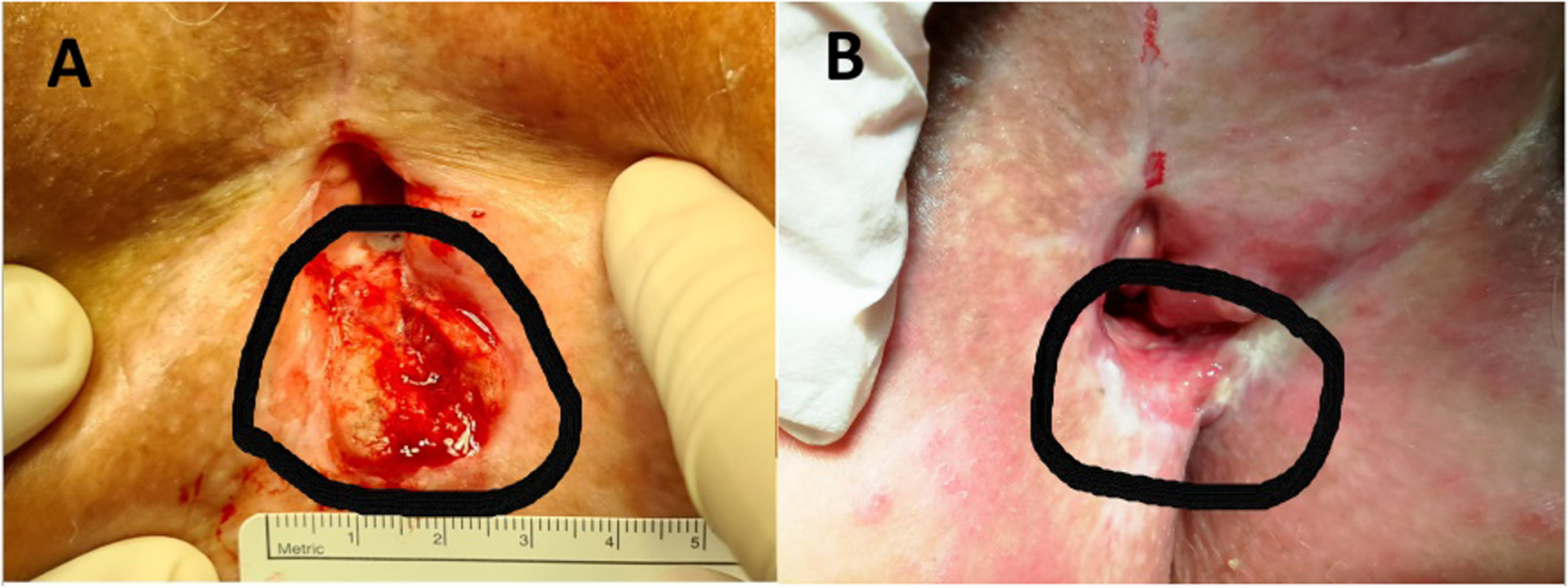
(A) Local recurrence of vulvar cancer (circled line) in the perineal region 10 months after primary treatment. (B) Complete response 12 months after treatment with electrochemotherapy (circled line).

The patient with recurrent vulvar cancer involving the perineum was treated with electrochemotherapy at the age of 64 years. After primary treatment with surgery and chemoradiotherapy, the patient was classified as FIGO stage IIIC vulvar cancer. We observed a local recurrence in the perineal region 10 months after primary treatment (Figure 1). Electrochemotherapy treatment of vulvar cancer perineum lesions was performed according to the European Standard Operating Procedures for Electrochemotherapy (ESOPE).^15^ Electrochemotherapy was performed under general anesthesia. The patient had no pain (0/10 on the visual analog scale [VAS]), fever, or malaise after treatment and was discharged from the hospital on the third day after the procedure. The patient was hospitalized for 4 days, compared with pelvic exenteration patients, in whom the average hospital stay was 19.75 (± 1.68) days. Two to 3 weeks after electrochemotherapy, the patient complained of pain, which was described as VAS 4/10. The pain was controlled with oral opioid and nonopioid analgesics. Twelve months after treatment with electrochemotherapy, there were no visible signs of disease progression in the vulvar region, and the lesions had a complete response (Figure 1). The patient had no problems with urination and defecation, no pain, and her quality of life was not affected after electrochemotherapy.

## Discussion

Pelvic exenteration in women with recurrent vulvar cancer in the perineal region is associated with high morbidity and mortality and substantial treatment costs.^12^ Because of prior radiation, surgery is often the only treatment option for local recurrent vulvar cancer in the perineal region, that is, exenterative surgical procedures with the formation of a bowel and/or urinary stoma.^27^ Centers tend to be reluctant and cautious in performing such pelvic surgeries. Exenterative surgery is chosen only for patients for whom there are no other treatment options and who are considered to benefit maximally from exenteration.^28^

Various reconstructive techniques have been proposed to fill the empty space after pelvic exenteration, including procedures using the omentum, absorbable meshes, or silicone expanders. In addition, vertical (VRAM) and transverse (TRAM) rectus abdominis myocutaneous flaps with vascular supply from branches of the deep inferior epigastric vessels have been successfully used for vaginal reconstruction after pelvic exenteration.^29–32^ The formation of bowel or urinary stoma can seriously affect social relations. This inconvenience is more critical in younger patients and in patients with recurrent diseases.^33,34^ An alternative to 2 separate stomas is double-barreled wet colostomy (DBWC). This is a lateral loop colostomy that contains both urinary and intestinal diversions in the same segment and drains through a single stoma. DBWC has a less negative impact on patient quality of life than 2 separate stomas.^35,36^

Spreading to the lymph nodes seems to be the most important prognostic factor, which has already been shown in other clinical and clinicopathologic studies. Moreover, there are no long-term survivors among patients with positive lymph nodes after pelvic exenteration.^2,37–39^ Therefore, the management of patients with recurrent local vulvar carcinoma in the perineal region remains a clinical dilemma, especially considering that these patients are often elderly and have many age-related comorbidities.

In our study, we proposed electrochemotherapy as another treatment modality that has been shown to be feasible and suitable for the treatment of recurrent vulvar cancer. Electrochemotherapy is a locoregional antitumor treatment. Two chemotherapeutic agents are currently used in electrochemotherapy, bleomycin, and cisplatin. Due to the wider use of bleomycin in electrochemotherapy protocols for different types of cancers, we decided also to use bleomycin for the treatment of recurrent vulvar cancer. However, based on our experience with electrochemotherapy with cisplatin of cutaneous tumor nodules in patients with malignant melanoma, squamous cell carcinoma, and basal cell carcinoma, cisplatin could also be effectively used for the treatment of superficial tumors.^40,41^ The efficacy of electrochemotherapy for skin and subcutaneous tumors is well established and represents a valuable alternative or complementary option for patients with superficial tumors.^42^ Treatment with electrochemotherapy has been shown to improve patients quality of life of patients and reduce symptoms such as bleeding, pain, odor, itching, and sexual dysfunction. In women with recurrent vulvar cancer, electrochemotherapy appears to be well tolerated and may lead to noticeable tumor control with concomitant symptom relief.^42^

Patients treated with electrochemotherapy had a shorter hospital stay and quality of life was not impaired after electrochemotherapy, compared with patients after pelvic exenteration in whom quality of life decreased significantly due to short- and long-term complications. In addition, one study found that women who underwent pelvic exenteration for gynecologic malignancies became more obese and comorbid during the study period, resulting in more complications, longer length of stay, and higher treatment-related costs. These data help define changes and trends in the use and outcomes of pelvic exenteration for gynecologic cancers.^12^

Unfortunately, we do not have data on the sexual quality of life of our patients. Little data and information on sexual quality of life have been available in the literature. Approximately 25% of women treated with vagina-sparing pelvic exenteration were sexually active and had low and moderate levels of intercourse pleasure. In this context, it seems reasonable to hypothesize that sparing the vagina as much as possible during supra levator pelvic exenteration might help to satisfy these unmet needs.^10^

Despite the relevance of our data, several limitations of the study should be noted. The main limitation of our study is the lack of validated quality of life questionnaires. Our data were collected from clinical records and do not allow definitive conclusions. The other limitation of the study is its retrospective observational nature.

However, because of the rare vulvar cancer recurrence in the perineal region and the even smaller number of physically fit patients willing to undergo such a radical surgical procedure, conducting a prospective randomized study to investigate the benefits of pelvic exenteration is challenging. Therefore, even small retrospective studies are important to verify the value of pelvic exenteration as the ultimate treatment for vulvar cancer. Most authors agree that pelvic exenteration should be performed only when there are no less radical alternatives or the option of radiation therapy.^28^

## Conclusions

Our experience showed that electrochemotherapy might be a less radical alternative to pelvic exenteration, especially for patients with initially higher FIGO stages.
